# Musculoskeletal Modeling of the Lumbar Spine to Explore Functional Interactions between Back Muscle Loads and Intervertebral Disk Multiphysics

**DOI:** 10.3389/fbioe.2015.00111

**Published:** 2015-08-05

**Authors:** Themis Toumanidou, Jérôme Noailly

**Affiliations:** ^1^Institute for Bioengineering of Catalonia, Barcelona, Spain; ^2^Department of Information and Communication Technologies, Universitat Pompeu Fabra, Barcelona, Spain

**Keywords:** constitutive muscle model, lumbar spine finite element model, intervertebral disk swelling, intervertebral disk–muscle interaction, standing, night rest

## Abstract

During daily activities, complex biomechanical interactions influence the biophysical regulation of intervertebral disks (IVDs), and transfers of mechanical loads are largely controlled by the stabilizing action of spine muscles. Muscle and other internal forces cannot be easily measured directly in the lumbar spine. Hence, biomechanical models are important tools for the evaluation of the loads in those tissues involved in low-back disorders. Muscle force estimations in most musculoskeletal models mainly rely, however, on inverse calculations and static optimizations that limit the predictive power of the numerical calculations. In order to contribute to the development of predictive systems, we coupled a predictive muscle model with the passive resistance of the spine tissues, in a L3–S1 musculoskeletal finite element model with osmo-poromechanical IVD descriptions. The model included 46 fascicles of the major back muscles that act on the lower spine. The muscle model interacted with activity-related loads imposed to the osteoligamentous structure, as standing position and night rest were simulated through distributed upper body mass and free IVD swelling, respectively. Calculations led to intradiscal pressure values within ranges of values measured *in vivo*. Disk swelling led to muscle activation and muscle force distributions that seemed particularly appropriate to counterbalance the anterior body mass effect in standing. Our simulations pointed out a likely existence of a functional balance between stretch-induced muscle activation and IVD multiphysics toward improved mechanical stability of the lumbar spine understanding. This balance suggests that proper night rest contributes to mechanically strengthen the spine during day activity.

## Introduction

Though statistics vary among different epidemiological studies, low-back pain (LBP) is one of the major health problems in industrialized countries (Podniece, 2008), affecting about half of the working population in Europe each year (Eurofound, 2012). Such incidence may even rise up to 90% depending on both the population studied and the definition adopted for LBP (Op de Beek and Hermans, 2000). Importantly, these epidemiological studies highlight the impact of occupational activities on the percentage of individuals suffering from symptomatic spine disorders.

Although the mechanisms that lead to LBP are complex and remain unclear, lumbar intervertebral disk (IVD) degeneration is a primary cause of back symptoms, such as muscle spasms (Frymoyer et al., 1984; Boden et al., 1990).). Multivariable analyses for known contributors of disk degeneration including genetic (Battié et al., 2009) and occupational factors (Riihimäki, 1991) revealed that over 50% of occurrences and progression of disk degeneration remains unexplained in the lower spine (L4–S1 levels) (Battié et al., 1994). Unidentified factors are likely to involve complex mechanobiological and multiphysics interactions in the IVD (Hsieh and Yoon, 2010), under the influence of the external mechanical loads transferred to the spine tissues. However, load transfers through muscles remain largely underexplored even for the most common sedentary postures/activities. Meanwhile, muscle weakness is thought to be linked with LBP (Heydari et al., 2010) but the extent which this link implicates biomechanical factors is unclear. Therefore, to understand the link between muscle activity and internal load transfer in low-back disorder, a first step would be to investigate the possible couplings between muscle activation and IVD mechanics in the healthy spine.

Due to the level of invasiveness associated with mechanical measurements in the lumbar spine, biomechanical models offer important investigative tools (Noailly and Lacroix, 2012). Inverse dynamics calculations, coupled to rigid body (RB) models, can be used to estimate the effect of the muscle function on non-deformable intersegmental articulations, from specific kinematical measurements (Han et al., 2013). Muscle forces can also be estimated by coupling RB models to Hill-type actuators for the muscles (Christophy et al., 2012), which allows combining inverse dynamics and static optimization methods while assuming minimization of muscle activation. Moreover, models based on the finite element (FE) method used kinematics-driven optimizations that take into account the non-linear passive resistance of the intervertebral joints (Arjmand et al., 2009; Gagnon et al., 2011). However, a shared limitation to these methods stems from calculating static muscle forces through which the time-dependent transient response of the IVD is difficult to reflect.

Clearly, one step forward would consist in considering constitutive muscle models that link together muscle forces and current deformations through unique sets of parameters, and can be coupled to spine FE models. For the cervical spine, such an approach allowed predicting different distributions of neck muscle loads produced during impact-induced motions (Hedenstierna and Halldin, 2008). Yet, to our knowledge, no such predictive models have been explored for the lumbar spine.

The present work proposes a new integrated FE musculoskeletal model of the lumbar spine where the muscle response was coupled to the mechanical behavior of the passive lumbar spine tissues. A constitutive model was proposed for the active and passive behavior of the major muscle groups that act on the lower back, and was coupled to a geometrical description of the corresponding fascicles in a L3–S1 lumbosacral FE model. Our specific objective was to explore the functional interaction between muscle function and the transient behavior of the osteoligamentous spine, largely controlled by IVD multiphysics in the healthy spine during daily activities. Since light intensity activities involving static and lying postures are suggested to determine most of the daily physical activity in normal population (Tikkanen et al., 2013), we focused on the simulation of standing and lying (night rest) positions.

## Materials and Methods

### Muscle model anatomy

A muscle architecture of 23 sagittally symmetric fascicle pairs was developed and featured three main muscle groups with insertions in the lumbar region: the multifidus (MF) and the erector spinae (ES) from the intrinsic muscles of the back, and the psoas major (PS). The bony insertions of the fascicles were adapted to the specific anatomy of the bi-segment lumbar spine model of Noailly et al. (2007), which was extended to include the L5/S1 intervertebral joint. The sagittal balance of the lumbosacral joint was proportionally related to the L4/L5 lordosis of the model according to reported anatomical measurements (Bogduk et al., 1992a).

Following the anatomical description from Bogduk (2005) (Chapter 9), the ES was divided into two muscle groups: the longissimus thoracis (LT) and the iliocostalis lumborum (IL) each of which was further divided into a lumbar and a thoracic component, according to the attachment points of the fascicles. As such, the lumbar and thoracic parts of the LT were defined by the Longissimus Thoracis pars Lumborum (LTpL) and the Longissimus Thoracis pars Thoracis (LTpTh), respectively. As for the IL, its lumbar part was the IL pars Lumborum, whereas its thoracic part has no attachment in the lumbar region and was not considered.

**Figure 1:**
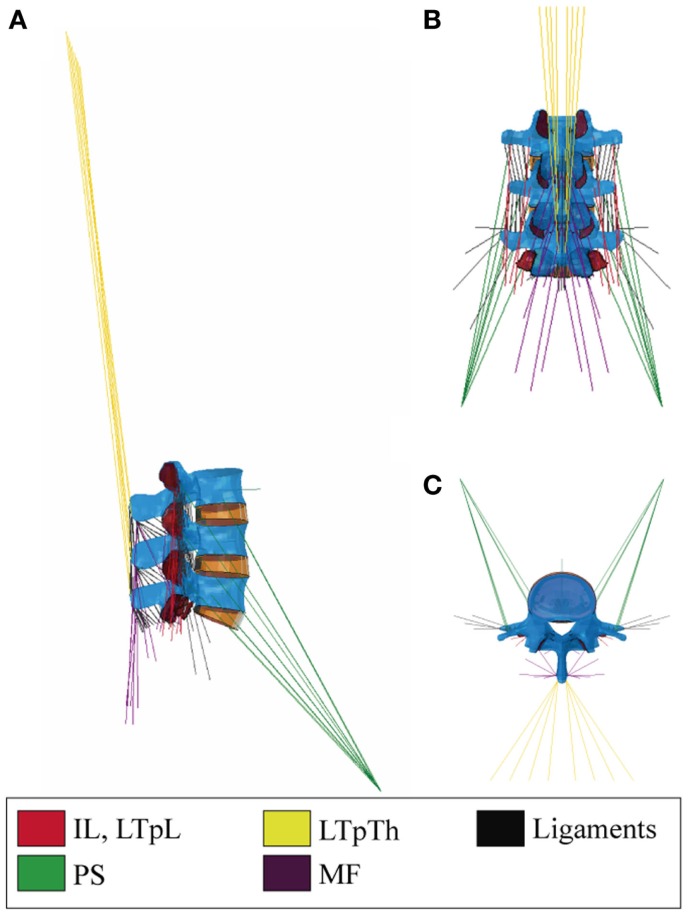
**(A)** Right sagittal, **(B)** back, and **(C)** top transverse views of the 46-muscle L3–S1 finite element model: Iliocostalis (IL) and Longissimus Thoracis pars Lumborum (LTpL), Longissimus Thoracis pars Thoracis (LTpTh), Multifidus (MF), Psoas (PS).

The LTpL and the IL were modeled with three and two symmetric fascicle pairs, respectively (Figure 1). The LTpTh was modeled with four symmetric fascicles with caudal insertions at the L3–L5 vertebrae and cranial insertions reconstructed to simulate the lines of action that virtually reach the T3–T6 levels of the thorax. We assumed a common rostral insertion of these cranial ends at the dorsal part of the third rib, represented as an enlarged transverse process of the third thoracic vertebra and modeled as a rigid rod. In simulated standing, this thoracic insertion was assumed to be cranio-caudally aligned with the uppermost vertebral level of the model, i.e., L3. A common musculotendon rest length was assumed for all thoracic elements based on the lumbar musculoskeletal RB model implemented by Christophy et al. (2012).

The incorporation of the MF into the model followed the anatomical descriptions of Rosatelli et al. (2008) and Bogduk (2005) (Chapter 9). In total, eight symmetric pairs of fascicles were included with caudal insertions at the lower lumbar levels and the sacrum. Cranial insertions were distributed from L3 to L5, and the fascicles formed three anteroposterior groups, for the deep, the intermediate and the superficial fascicles, respectively. As for the PS, we adopted the description from Bogduk et al. (1992b) and Bogduk (2005) (Chapter 9) and incorporated six overlapping segmental fascicles on each side, between the anterolateral aspects of the vertebra and the lesser trochanter of the femur (Figure 1). Approximation of the common femoral point of insertion was based on the musculoskeletal models reported in several studies (Delp et al., 2007; Christophy et al., 2012; Sánchez Egea et al., 2014).

Overall, the muscle network consisted of 13 pairs of local fascicles (attached to lumbar vertebra) and 10 pairs of global fascicles (attached to ribcage), all modeled as unidirectional elements with straight lines of action (Figure 1). Fascicle 3D orientations and equivalent cross-sectional areas, i.e., area calculated by dividing the fascicle volume by the fascicle length, were derived from anatomic studies and radiographic measurements as reported by Bogduk et al. (1992a,b). In particular, the axial and posterior lines of actions of the ES fascicles were resolved in the local coordinate system of the vertebra according to Bogduk et al. (1992a) (Supplementary Material).

### Muscle constitutive model

The muscle constitutive model assumed that the respective strain energies of the matrix and the embedded muscle fibers could be decoupled (Weiss et al., 1996). The tissue was regarded as a fiber-reinforced composite material. Fibers were modeled based upon the three-element description initially proposed by Hill (1938) for skeletal muscles that included parallel (PE), series (SE), and contractile (CE) elements. The mathematical representation of the respective contributions of these elements was inspired from the work of Martins et al. (1998), and is further described through Eqs 5–9.

For the matrix, the dilatational (*U*_J_) and deviatoric (*U*_I_) strain energy densities were also decoupled (Eq. 1). A Neo-Hookean formulation was used for *U*_I_ (Eq. 2). For *U*_J_, we used the definition proposed by Weiss et al. (1996) (Eq. 3). All in all, the muscle was modeled as an active, transversely isotropic and hyperelastic solid, the strain energy of which was given by:

(1) $$U=U_{\text{I}}\left({\bar{\text{I}}}_{1}^{\text{C}}\right)+U_{\text{J}}\left(J\right)+U_{\text{F}}\left({\bar{\lambda }}_{\text{f}},\zeta^{\text{CE}}\right)$$

where

(2) $$U_{\text{I}}\left({Ī}_{1}\right)=\frac{G}{2}\left({\bar{\text{I}}}_{1}^{\text{C}}-3\right)$$

is the strain energy associated with the deviatoric response of the matrix.

(3) $$U_{\text{J}}\left(J\right)=\frac{K}{2}\ln{\left(J\right)}^{2}$$

is the strain energy associated with the volume change, and

(4) $$U_{\text{F}}\left({\bar{\lambda }}_{\text{f}}\text{, }\zeta^{\text{CE}}\right)=\sigma_{0}\int_{\text{1}}^{{\bar{\lambda }}_{f}}f_{\text{PE}}\left(\lambda \right)d\lambda +\sigma_{0}\;\int_{\text{1}}^{{\bar{\lambda }}_{\text{f}}}f_{\text{SE}}\;\left(\lambda \text{, }\zeta^{\text{CE}}\right)d\lambda$$

is the strain energy stored in the muscle fibers.

In Eqs 1–3, *J* is the Jacobian determinant of the deformation gradient **F**, *G* is the matrix shear modulus, ${\bar{\text{I}}}_{1}^{\text{C}}$ is equal to *J*^−2/3^ tr**C**, i.e., the first invariant of the deviatoric part of the right Cauchy–Green strain tensor **C**, and *K* is the matrix bulk modulus. In Eq. 4, ${\bar{\lambda }}_{\text{f}}$ is equal to $\sqrt{N^{T}\bar{C}N}$, **N** being the orientation vector of the fiber in the undeformed fascicle, and $\bar{C}$ the deviatoric part of **C**. In other words, ${\bar{\lambda }}_{\text{f}}$ is equivalent to $J^{{}^{-1}\;\;{/}_{\;\;3}\;}\lambda$, where λ is the longitudinal fascicle stretch ratio. σ_0_ is the maximum tetanic stress, and ζ^CE^ is the contraction amplitude reflecting the muscle activation level of the CE: this parameter is further described in Eq. 9.

When non-activated, stretched muscles produced a positive fiber stress that developed only in the PE branch of the rheological model:

(5) $$\sigma^{\text{PE}}=\sigma_{0}f_{\text{PE}}\left({\bar{\lambda }}_{\text{f}}\right)$$

where

(6) $$f_{\mathrm{PE}}\left({\bar{\lambda }}_{f}\right)=\left\{\begin{array}{cc} A{\left({\bar{\lambda }}_{f}-1\right)}^{2},\; & \text{if }{\bar{\lambda }}_{f}>1\\ 0,\; & \text{otherwise} \end{array}\right.$$

The quadratic formulation of Eq. 6 was proposed by Chen and Zeltzer (1992) based on force-elongation measurements on frog muscles without activation, where *A* is a constant dimensionless material parameter.

When muscles were activated, a stress response was additionally produced in the SE of the active branch of the rheological model, in interaction with the CE. The overall active stress was given by:

(7) $$\sigma^{\text{SE}}\text{=}\sigma_{\text{0}}f_{\text{SE}}\left({\bar{\lambda }}_{\text{f}},\zeta^{\text{CE}}\right)$$

where

(8) $$f_{\mathrm{SE}}\left({\overset{¯}{\lambda }}_{f},\zeta^{\text{CE}}\right)=\left\{\begin{array}{cc} 0.1\left\{\exp\left[100\left({\overset{¯}{\lambda }}_{f}-1-\zeta^{\text{CE}}\right)\right]-1\right\},\; & \text{if }{\bar{\lambda }}_{f}>1+\zeta^{\text{CE}}\\ 0,\; & \text{otherwise} \end{array}\right.$$

is the contractile stress-stretch function. The non-zero expression of Eq. 8 represents the muscle response at the ascending (concentric) or descending (eccentric) limb of the active tension-length curve, depending on the value of the strain-like parameter ζ^CE^. The latter can be decoupled as:

(9) $$\zeta^{\text{CE}}=\frac{L^{\text{CE}}-L_{\text{0}}^{\text{CE}}}{L_{\text{0}}^{\text{M}}}=\frac{L_{\text{0}}^{\text{CE}}}{L_{\text{0}}^{\text{M}}}\left(\frac{L^{\text{CE}}-L_{\text{0}}^{\text{CE}}}{L_{\text{0}}^{\text{CE}}}\right)=C_{\text{CE}}\cdot \varepsilon$$

ζ^CE^ is proportional to the engineering strain $\varepsilon =\left(\frac{L^{\text{CE}}-L_{\text{0}}^{\text{CE}}}{L_{\text{0}}^{\text{CE}}}\right)$, and avoids explicit input of the activation level to describe active contraction in the CE, according to the phenomenological approach reported by Martins et al. (1998). The parameter controls the level of stretch-induced fascicle activation through the ratio $\frac{L_{\text{0}}^{\text{CE}}}{L_{\text{0}}^{\text{M}}}$, hereafter called active parameter *C*_CE_, where $L_{\text{0}}^{\text{M}}$ and $L_{\text{0}}^{\text{CE}}$ are the optimal and the resting fascicle lengths, respectively. Given that no information could be retrieved from the literature for $L_{\text{0}}^{\text{M}}$, we adopted the approximation proposed by Delp et al. (2001), and considered the length ratio $\frac{L_{\text{0}}^{\text{CE}}}{L_{\text{0}}^{\text{M}}}$ equivalent to the ratio between optimal and resting sarcomere lengths. Hence, we calculated *C*_CE_ by normalizing the sarcomere length estimations, *L*^S^, reviewed by Christophy et al. (2012) with an optimal sarcomere length $L_{\text{0}}^{\text{S}}$ equal to 2.8 μm (Walker and Schrodt, 1974; Lieber et al., 1994):

(10) $$L_{\text{0}}^{\text{M}}=L_{\text{0}}^{\text{CE}}\times \frac{L_{\text{0}}^{\text{S}}}{L^{\text{S}}}\Leftrightarrow C_{\text{CE}}=\frac{L_{\text{0}}^{\text{CE}}}{L_{\text{0}}^{\text{M}}}=\frac{L^{\text{S}}}{2.8}$$

The value of *C*_CE_ was considered consistent among all fascicles of a given muscle group. *L*^S^ estimations for all muscle groups but the PS (Christophy et al., 2012) led to a first set of *C*_CE_ values lower than 1, as reviewed in Table 1. Such values, however, did not allow fulfilling the strain-based criteria to induce activation in our specific modeling framework (Eq. 8). Hence, a range of active parameter values for deformation levels up to 30% were based on a previous parametric analysis (Toumanidou et al., 2013), so as to satisfy Eq. 8 criteria for all the muscles modeled. As a result, a set of two *C*_CE_ values, i.e., *C*_CE1_ and *C*_CE2_ were obtained per muscle group (Eq. 11, Table 1) assuming that the relative activation from one muscle to another due to the morphometric differences (such as *L*^S^) should be preserved. *C*_CE1_ and *C*_CE2_ controlled the active behavior when the muscle was at the ascending and descending limb, respectively. As such, Eq. 9 was updated by:

(11) $$\zeta^{\mathrm{CE}}=\left\{\begin{array}{cc} C_{\text{CE1}}\cdot \varepsilon ,\; & \text{for}\;\varepsilon <0\;(\text{concentric})\\ C_{\text{CE2}}\cdot \varepsilon ,\; & \text{for}\;\varepsilon >0\;(\text{eccentric})\\ 0,\; & \text{otherwise} \end{array}\right.$$

**Table 1:** **Constitutive model parameters**.

PE parameters		Matrix parameters	
*A* (dimensionless)	4.0^a^	*G* (MPa)	16.42 × 10^−4c^
σ_0_ (MPa)	0.46^b^	*K* (MPa)	1.642

**CE parameters**	

		[D], [W], [Wb]^d^	Parametric study (*C*_CE1_*, C*_CE2_)^e^
*C*_CE_	MF	0.811	0.706/0.465
(dimensionless)	LTpL, LTpTh	0.825	0.718/0.473
	ILpL	0.846	0.737/0.485
	PS	1.111	0.967/0.637

*^a^Chen and Zeltzer (1992)*.

*^b^Average value of the reported range between 0.16 and 1 MPa (Zajac, 1989)*.

*^c^Adapted from Martins et al. (1998)*.

*^d^Calculated based on ([D]: Delp et al. (2001); [W]: Ward et al. (2009a) and [Wb]: Ward et al. (2009b))*.

*^e^Values calculated for fascicle shortening (concentric contraction)/fascicle elongation (eccentric contraction) (Toumanidou et al., 2013)*.

The model considered the velocity of deformation of the CE as the time derivative of the parameter ζ^CE^ (Eq. 11) (Martins et al., 1998; Ho Ba Tho et al., 2014), here implicitely considered through the rate of ε change along the simulations. According to Eq. 8, the maximum contraction velocity (*v*_max_) corresponding to *f*_SE_ = 0 (Hill, 1938) was reached when ${\bar{\lambda }}_{\text{f}}$ was equal to 1 + ζ^CE^, which depended on the strain ε, and on the muscle group via *C*_CE1_.

Table 1 summarizes all the muscle model parameter values. σ_0_ varies both from species to species and subject to subject, but no values have been reported particularly for the back muscles so far. As such, a value of 0.46 MPa was chosen lying in the mean of the reported range for skeletal muscles (0.16–1 MPa) (Zajac, 1989). As for *K*, a nearly incompressible matrix was simulated (Blemker et al., 2005), and since no specific values were available, we prescribed *K* equal to 1000 times *G* (Weiss and Gardiner, 2001), the value of *G* being based on Martins et al. (1998). Setting *A* equal to 4.0 allowed best fit of model predictions to experimental measurements on frog skeletal muscles (Chen and Zeltzer, 1992). Given the similar striated form of human and frog skeletal muscles, the latter value was adopted, as previously proposed by others (Martins et al., 1998; Lu et al., 2011).

Finally, using Eqs 1–4, the second Piola–Kirchhoff stress tensor ***S*** in the muscle tissue was obtained from the strain energy function of Eq. (1):

(12) $$S=2\frac{\partial \text{U}}{\partial \text{C}}=\frac{G}{2}\left(2J^{-2∕3}I-\frac{2}{3}{\bar{\text{I}}}_{1}^{\text{C}}C^{-1}\right)+\mathrm{lnJ}\;C^{-1}+U\prime_{\text{F}}\left[J^{-\frac{2}{3}}{\bar{\lambda }}_{\text{f}}^{-1}\left(N⊗N\right)-\frac{1}{3}{\bar{\lambda }}_{\text{f}}C^{-1}\right]$$

where **C** is the right Cauchy–Green strain tensor, and:

(13) $$U\prime_{\text{F}}=U\prime_{\text{PE}}\left({\bar{\lambda }}_{\text{f}}\right)+U\prime_{\text{SE}}\left({\bar{\lambda }}_{\text{f}},\zeta^{\text{CE}}\right)$$

with

(14) $$U\prime_{\text{PE}}\left({\bar{\lambda }}_{\text{f}}\right)=\sigma_{\text{0}}f_{\text{PE}}\left({\bar{\lambda }}_{\text{f}}\right)$$

(15) $$U\prime_{\text{SE}}\left({\bar{\lambda }}_{\text{f}},\zeta^{\text{CE}}\right)=\sigma_{\text{0}}f_{\text{SE}}\left({\bar{\lambda }}_{\text{f}},\zeta^{\text{CE}}\right)$$

The Cauchy stress was related to the second Piola–Kirchhoff stress by:

(16) $$\sigma =\frac{1}{J}\;{\mathrm{FSF}}^{-\text{T}}=\frac{G}{2}\left(2{\bar{C}}^{-T}-\frac{2}{3}{\bar{\text{I}}}_{1}^{\text{C}}I\right)+\frac{\mathrm{lnJ}}{J}I+\frac{1}{J}\left[U\prime_{\text{F}}\left({\bar{\lambda }}_{\text{f}}\left(n⊗n\right)-\frac{1}{3}{\bar{\lambda }}_{\text{f}}I\right)\right]$$

where ***n*** is the direction of the muscle fibers in the deformed fascicle, and **I** is the second-order unit tensor.

### Coupled lumbar FE muscle model and material properties

The constitutive model was coupled to the L3–S1 musculoskeletal spine FE model by means of user-defined material subroutines (UMAT) and an implicit solver was used. Geometrical details for the vertebrae, IVDs, and facet cartilages were previously reported (Noailly et al., 2007), as well as the mesh refinement used for proper disk convergence during poromechanical analyses (Ruiz et al., 2013).

Hypoelastic formulations were considered for all ligaments with the geometrical properties based on Noailly et al. (2011) (supraspinous, interspinous, ligamentum flavum, capsular, intertransverse, posterior, and anterior longitudinal) and Aihara et al. (2002) (iliolumbar). Ligament material parameters were calculated by Noailly et al. (2011) from literature experimental data. Poroelastic IVD models were considered, including osmo-porohyperelastic and fiber-reinforced porohyperelastic laws for the Nucleus Pulposus (NP) and the annulus fibrosus (AF), respectively (Malandrino et al., 2009, 2011, 2013) (Table 2).

**Table 2:** **IVD material parameters**.

Material	*G* (MPa)	*K* (MPa)	*e*_0_^d^	k_0_ (mm^4^/N⋅s)^e^	*M*
Annulus fibrosus	0.95^a^	0.37^b^	3.0^b^	0.0002^b^	8.5^b^
Nucleus pulposus	0.47^a^	0.16^b^	4.9^b^	0.0009^b^	8.5^b^
Cartilage endplate	8.55^a^	10.10^b^	4.0^b^	0.0025^b^	8.5^b^
Strain-dependent permeability^c^: $k=k_{0}{\left[\frac{e\left(1-e_{0}\right)}{e_{0}\left(1+e\right)}\right]}^{2}\exp\left[M\left(\frac{1+e}{1+e_{0}}-1\right)\right]$					

*^a^Malandrino et al. (2009)*.

*^b^Malandrino et al. (2011)*.

*^c^Argoubi and Shirazi-Adl (1996)*.

*^d^Initial void ratio*.

*^e^Initial permeability*.

### Loading cases and boundary conditions

**Figure 2:**
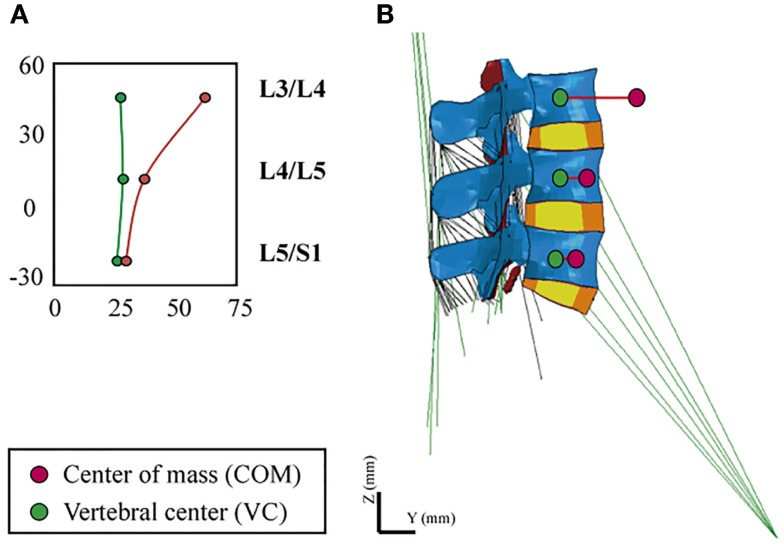
**(A)** Definition of the eccentric gravity load path passing through the segmental COM, **(B)** VC and COM locations at the L3–S1 FE model.

In order to simulate the standing position, we took into account the heterogeneous distribution of body volumes and densities along the trunk (Pearsall et al., 1996; Vette et al., 2011). We calculated the magnitude and point of application of an equivalent gravity load per simulated level, i.e., we translated the body mass distributions into punctual static loads, in function of the contribution expected from the rest of the upper body. In order to place the vertical loads, we defined an eccentric path that passed through the different segmental centers of mass (COM), anterior to the vertebral center (VC), and calculated the posteroanterior distance between VC and COM, *R*_i_, per level (Figure 2). Calculations were applied to simulate a body weight of 70.8 kg of a normal subject.

For the loads associated to the weight of the head and of the cervical spine (C1–C7), we defined the percentages of body mass (BM_i_) with respect to the total body weight based on Ivancic et al. (2006). While we used directly the mass moment of inertia, Iz_i_ (where z is the axial direction), values reported by the authors, we recalculated the BM_i_ values proportionally to our simulated body mass. For the loads induced by the body mass in the thoracic (T1–T12) and lumbar (L1–S1) regions, we recalculated the BM_i_, relative to our assumed body weight, and we adapted the Iz_i_ values to these BM_i_, in function of the *R*_i_ values reported by Pearsall et al. (1996). All BM_i_ and Iz_i_ values used for the calculation of the effective loads over the L3–S1 model are summarized in Table 3.

**Table 3:** **Sagittal moment of inertia, moment arm, and mass properties**.

*i*	Body mass (%)^a^	Mass (kg)^a^	Iz_i_, tot (kg⋅cm^2^)^a^	
HD	4.7	3.300	1.60E + 02	
C1	0.6	0.404	0.63E + 00	
C2	0.7	0.508	1.10E + 00	
C3	0.5	0.363	0.45E + 00	
C4	0.5	0.366	0.47E + 00	
C5	0.5	0.371	0.49E + 00	
C6	0.6	0.439	0.69E + 00	
C7	0.7	0.505	1.19E + 00	

***i***	**Body mass (%)^b^**	**Mass (kg)^b^**	**Iz_i_ (kg⋅cm^2^)**	**Iz_i_, _L3_ (kg⋅cm^2^)**

T1	1.1	0.811	4.98E-01	4.49E + 00
T2	1.1	0.780	1.32E + 00	4.49E + 00
T3	1.4	0.976	3.96E + 00	4.80E + 00
T4	1.3	0.920	7.22E + 00	3.68E + 00
T5	1.3	0.945	1.06E + 01	2.66E + 00
T6	1.3	0.932	1.39E + 01	1.33E + 00
T7	1.4	0.976	1.83E + 01	4.86E-01
T8	1.5	1.049	2.22E + 01	1.70E-01
T9	1.6	1.096	2.39E + 01	0.11E-01
T10	2.0	1.419	2.99E + 01	0.57E-01
T11	2.1	1.479	2.88E + 01	3.70E-01
T12	2.5	1.767	2.97E + 01	8.67E-01
L1	2.4	1.677	2.08E + 01	6.12E-01
L2	2.4	1.689	1.24E + 01	2.72E-01
L3	2.3	1.670	5.27E + 00^c^	–
L4	2.6	0.180	2.18E-01	–
L5	2.6	0.182	2.92E-02	–

*^a^Ivancic et al. (2006)*.

*^b^Pearsall et al. (1996)*.

*^c^The Iz at L3/L4 here does not refer to the Iz_,eff_ used in the calculations in Eq. 18 in the manuscript. The value of Iz at L3/L4 is the local Iz reported for calculations of full L1–S1 models (in this case, Iz_,eff_ was calculated at L1–L2 level)*.

In order to calculate an effective moment of inertia, Iz_,eff (L3)_, at L3/L4, we considered the Huygens–Steiner theorem for all the upper thoracic (T1–T12) and lumbar (L1–L3) levels: we calculated the moment of inertia, Iz_i,L3_, of each of these levels, i, with respect to the vertical axis, COM_3_, passing through the L3/L4 COM. As such, Iz_i,L3_ was the product of the body mass of level i with the square of the perpendicular distance, *d*_i_, between the axis COM_3_, and the vertical axis, COM_i_, that passes through the COM of level i (Table 3):

(17) $$\text{I}{\text{z}}_{\text{i,L3}}\;=\text{ B}{\text{M}}_{\text{i}}{d_{\text{i}}}^{\text{2}}$$

All COM_i_ axes were adapted to our model geometry based on the measurements reported by Pearsall et al. (1996). Despite a thorough literature review, no relevant *d*_i_ values could be found for the head and cervical spine levels. As such, we considered that the cervical curvature is only slightly moved in the anteroposterior direction from the L3/L4 lordotic angle. Thus, at the cervical levels, the squared perpendicular distance $d_{\text{i}}^{2}$ would be of the order-of-magnitude of −2, and according to Eqs 16 and 17, the contribution of these levels on Iz_,eff (L3)_ could be neglected. Overall, the total moment of inertia at L3/L4 level, Iz_,eff (L3)_, was the sum of 14 Iz_i,L3_ contributions (from T1–T2 to L2–L3) plus the local moment of inertia, Iz_3_, at L3/L4 with respect to COM_3_:

(18) $$\text{I}{\text{z}}_{\text{,eff(L3)}}=\sum_{\text{T1}}^{\text{L3}}I_{\text{Zi,L3}}+I_{{\text{Z}}_{\text{3}}}$$

Finally, through the resultant Iz_i_ and BM_i_ values, we estimated the effective distance *R*_eff (L3)_ at which the effective gravity load has to be applied at L3/L4, in order to take into account those superior levels not included in the L3–S1 model. For the lumbar levels caudal to L3, i.e., from L4 to S1, local boundary loads were simply defined by using the BM_i_ and Iz_i_ values derived from the data reported by Pearsall et al. (1996), as described above (Table 3). All in all, a total gravity load of 276 N was distributed as follow:

- 239 N at 41.4 mm anterior to the segmental VC at the L3/L4 level [Iz_,eff (L3)_]
- 18.1 N at 11 mm anterior to the segmental VC at the L4/L5 level
- 18.1 N at 4 mm anterior to the segmental VC at the L5/S1 level.

In order to simulate the lying position, we considered a free IVD swelling condition due to an initial gradient of osmotic pressure of 0.15 MPa between the NP and the IVD model boundaries (Johannessen and Elliott, 2005). This swelling was simulated for a period of 8 h and aimed to mimic overnight rest. For the standing position, we applied the distributed gravity load of 276 N in 60 s, (a) without and (b) with previous night rest.

**Figure 3:**
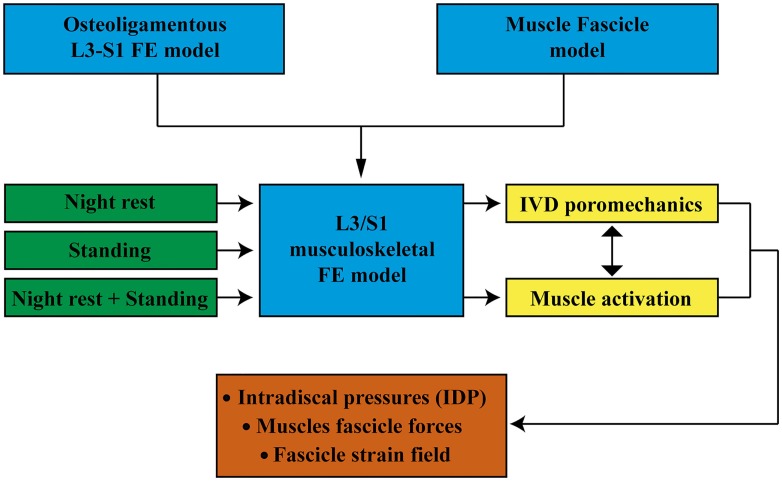
**Schematic diagram of the model input and output and the simulations carried out**.

For all simulations, the lower endplate of the L5/S1 IVD as well as the upper facets of S1 and the sacral and pelvic muscle insertions were fixed in all directions. External pore pressure was nil at the external boundaries of all IVDs. All calculations were performed with the implicit FE solver Abaqus/Standard (6.12. Simulia, Providence, RI, USA). Muscle forces were predicted per group, per fascicle, and per level, and the relative contribution of the muscle model constitutive terms was calculated in both lying and standing positions. The effect of previous swelling on intra-level force distribution was quantified in simulated standing. We also calculated the intradiscal pressure (IDP) at the center of the NP (Supplementary Material) for all IVDs, and explored the effect of muscle activation on these calculations. Figure 3 shows a schematic diagram of the model input and output and the simulation cases discussed in this paper.

## Results

### Muscle forces and strains

**Figure 4:**
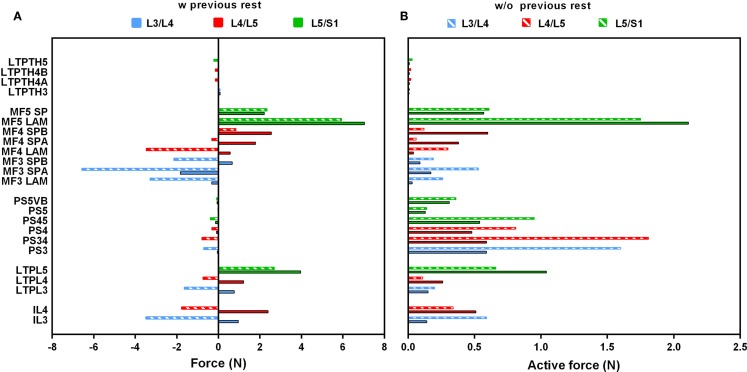
**(A)** Total force and **(B)** active force predictions per fascicle and per level in standing position with and without previous rest. On the vertical axis, the abbreviated name of the fascicles was informed by codifying the insertions: SP, spinous process; LAM, Lamina; VB, vertebral body. As for “A” and “B,” it simply says that the same fascicle has two components “A” and “B.”

In standing position without previously simulated rest, force calculations revealed activation of the PS and of all dorsal muscles, and a low contribution of the thoracic fascicles. At the upper levels (L3/L4, L4/L5), MF and IL fascicles transferred significant compression forces between nearly 3.5 and 6.5 N to the vertebrae and to the IVDs over which they span (Figure 4A). Among the local back muscles, the highest active forces were estimated for the MF fascicles arising from L5 (Figure 4B) that accounted for more than 1.5 N over a total of nearly 6 N force developed at this level. For the PS fascicles, the total compression forces developed were up to 1 N with relatively high contribution of positive active forces mainly in the upper region (Figure 4B). When previous rest was simulated, increased force activation was predicted for the caudal dorsal fascicles with positive total forces of up to about 7 N. For MF, active forces over 2 N were predicted at the lumbosacral level. For the global fascicles, PS fascicles were less activated at all levels and developed compression forces that did not exceed 0.13 N at L5/S1 level. Contribution of LTpTh remained low.

**Figure 5:**
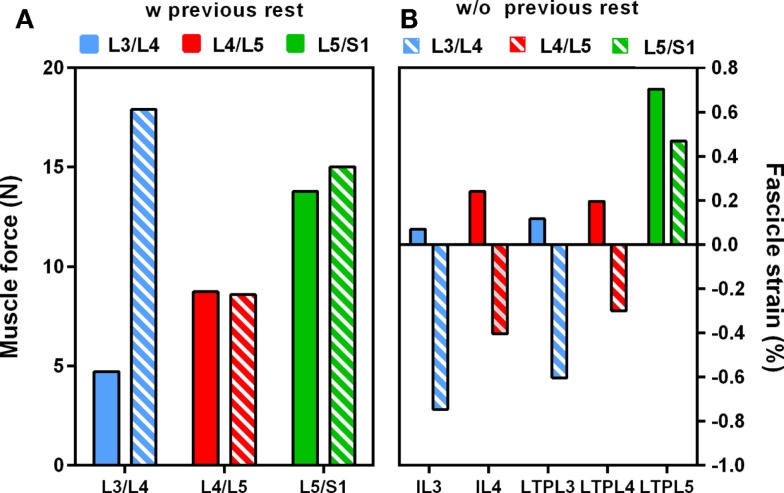
**Effect of swelling on (A) total intra-level force variation, (B) local muscle strains in standing position with and without previous rest**.

**Figure 6:**
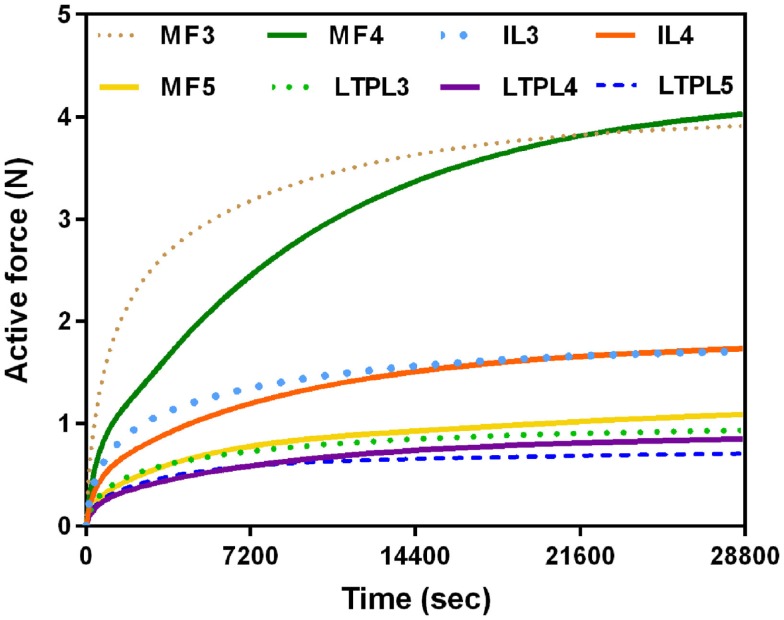
**Local muscle activation during night rest (8 h)**.

When previous lying was considered, muscle forces per level increased linearly in caudal direction in standing position. The maximum muscle resultant force was about 14 N at the lowermost L5/S1 level, i.e., nearly three times the total force calculated at L3/L4 (Figure 5A). Without previous lying, the maximum resultant force was approximately 18 N and was developed at L3/L4. Fascicle strain calculations in simulated standing showed that when previous lying was considered, most of the dorsal fascicles were stretched, whereas the bilateral fascicle contraction changed when no previous rest was considered (Figure 5B). Actually, along the IVD swelling simulated during the 8-h of rest (Figure 6), active forces were developed by the local muscles while the latter were stretched. As shown in Figure 6, L3 and L4 MF and IL fascicles were the most activated ones.

### Intradiscal pressure

**Figure 7:**
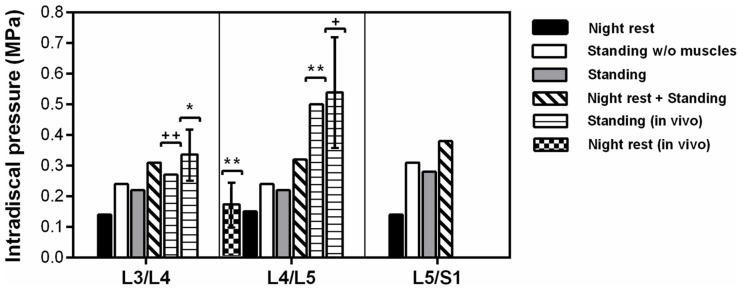
**Intradiscal pressure predictions per level at the center of NP and correlation with *in vivo* studies** [*Andersson et al. (1974); **Wilke et al. (1999); ^+^Sato et al. (1999)].

In standing position without previous rest, the IDP was 0.22 MPa at L3/L4 and L4/L5, while it was 0.28 MPa in the lumbosacral disk (Figure 7). Simulation of previous swelling increased the pressure in standing by 34–43% along the lumbar levels. The prediction was 0.31 MPa at L3/L4 where the *in vivo* measurement of Schultz et al. (1982) gave 0.27 MPa, and where the values measured by Andersson et al. (1974) ranged between 0.26 and 0.42 MPa. At L4/L5, the IDP calculated was 0.32 MPa and was in the range of the *in vivo* values between 0.22 and 0.75 MPa measured by Sato et al. (1999) (Table 4), though slightly below the inferior SD of the same measurements (Figure 7). Interestingly, calculations without muscles showed that inclusion of the latter contributed to decrease the pressure in standing position by up to 9% when previous rest was not simulated. During the 8-h of simulated rest, an overall pressure increase of 0.14 MPa was calculated at all levels, laying in the 0.10–0.24 MPa range of *in vivo* pressure increases reported over a period of 7 hours rest by Wilke et al. (1999) (Figure 7). The IDP results and previous *in vivo* measurements found in literature are summarized in Table 4.

**Table 4:** **Intradiscal pressure (MPa) predictions in the center of NP**.

	L3/L4	L4/L5	L5/S1
Standing	0.22	0.22	0.28
Standing w/o muscle function	0.24	0.24	0.31
Standing with previous rest	0.31	0.32	0.38
Night rest (overnight increase)	0.14	0.15	0.14
Standing *in vivo* (Schultz et al., 1982)^a^	0.27	–	–
Standing *in vivo* (Andersson et al., 1974)^b^	0.26-0.42	–	–
Standing *in vivo* (Sato et al., 1999)^c^	–	0.22–0.75	–
Standing *in vivo* (Wilke et al., 1999)^d^	–	0.50	–
Overnight increase *in vivo* (Wilke et al., 1999)^d^	–	0.10–0.24	–

*^a^Piezoresistive transducer, *n* = 4 subjects, mean 62.8 kg*.

*^b^Piezoresistive transducer, *n* = 4 subjects, mean 61.3 kg*.

*^c^Piezoresistive transducer-side window, *n* = 8 subjects, mean 73 kg*.

*^d^Piezoresistive transducer-implanted, *n* = 1 subject, 70 kg*.

## Discussion

In this paper, we proposed a new predictive lumbar spine muscle model that we used in order to explore the interaction between muscle function and IVD multiphysics for light muscle activities, such as those involving lying and relaxed standing. Although the components of the constitutive model have been already used in previous formulations (Martins et al., 1998; Blemker et al., 2005), the current model consists of a new assembly of these elements applied for the first time to the lower back muscles. The model was computationally efficient, which allowed its successful integration into a musculoskeletal FE model of the lower lumbar spine, as well as calculations of interactions between muscle loads and disk swelling. Though muscle force calculations were performed through unidirectional elements, our mechanical formulation considers the full Cauchy–Green strain tensor **C**. Hence, the mechanical law can be readily coupled to volumetric fascicle descriptions, whenever these descriptions become available.

The validation of the present musculoskeletal FE model represents an important challenge. In our study, the L3–L5 osteoligamentous FE model came from a previously validated model geometry (Noailly et al., 2007) and the adopted poroelastic IVD model was validated by Malandrino et al. (2013) against *in vitro* data (Heuer et al., 2008). The ligament formulation used has also shown its capacity to lead to the validation of different lumbar spine FE models (Noailly et al., 2007; Malandrino et al., 2015). However, the validation of our lumbar musculature model requires prediction assessments at the fascicle level through *in vivo* measurements of the muscle activity, which remains challenging. On one hand, the large number of deep fascicles, e.g., in the PS and the deepest layer of MF (shown to be influent mechanical components in the present manuscript), do not favor EMG registrations, as previously reported for intact back intrinsic muscles (Arjmand and Shirazi-Adl, 2006). Surface excitation/measurement techniques, such as elastography (Bensamoun et al., 2013), or assumption of PS activity on the basis of surface EMG signals recorded for the internal oblique (Cholewicki and McGill, 1996) would not be adequate given the statistical differences calculated especially in flexion tasks (McGill et al., 1996). On the other hand, in the latter study, the authors reported a difficulty in separating the EMG signals of adjacent muscles captured through intramuscular wires, e.g., between PS and Quadratus lumborum activity.

In general, direct measurement of muscle forces is complex, and previous measurements of the myoelectric activity of back muscles (Nachemson, 1966; Danneels et al., 2001; Stokes et al., 2003; Gagnon et al., 2011) did not allow reliable estimations of these forces. In the study of Brolin and Halldin (2005), comparisons between the predicted displacements and rotations during impact simulations for the cervical spine were reported against sled tests performed in human volunteers. Similar data do not exist for the lumbar spine to our knowledge, mainly due to the complex muscle anatomy of the lumbar zone. Alternatively, *in vivo* lumbar spine kinematics can be captured and serve for indirect estimations of particular muscle forces through kinematics-driven static optimizations. Analysis of related results reported by Arjmand et al. (2007) revealed that our relative force predictions among the different L4/L5 lumbar ES fascicles correlated well with the ES force distributions estimated by the authors for subjects with similar body weight in upright standing holding a 180 N load in their hands.

The scarcity of reported measurements, e.g., electrical activity of the modeled lumbar spine fascicles, does not benefit the creation of multi-scale constitutive laws including muscle electrophysiology, i.e., up to the cellular level, as previously proposed for lower limb muscles (Fernandez et al., 2005; Röhrle et al., 2012). We are aware that continuum-mechanical models cannot be used to investigate intrinsic properties of skeletal muscles, such as motor-unit recruitment or cross-bridge overlap. Indeed, multi-scale approaches have been proposed to include the effect of these features into continuum-based mechanical representation of muscle (Heidlauf and Röhrle, 2014). However, the complexity of the solving procedures, and the high calculation times make difficult the integration of such modeling approaches to advanced osteoligamentous spine models. Also, the representation of different types of fibers that influence the velocity of contraction would further increase the number of model parameters, the calibration of which would be largely speculative. In fact, Hill-type muscle models provide great insights for force-strain predictions on a larger scale, e.g., the lumbar musculoskeletal system (Hill, 1938; Winters and Stark, 1987). Hence, as a first approach we preferred was a phenomenological approximation based on a pragmatic modification of the Hill-type muscle model proposed by Martins et al. (1998), so as to extract the apparent effect of mechanical stretch on fascicle activation. Nevertheless, we acknowledge that this modification would require a thorough verification process through the simulation of well-documented muscles.

Importantly though, coupling our lumbar musculature model with geometrically and mechanically valid osteoligamentous components of the lumbar spine model allowed valuable assessments of the predicted muscle action. During previous rest, the slight axial spine distraction induced by the swelling of the IVDs stretched all the fascicles. This stretch rose up to 1.5% in the MF fascicles, which was enough to induce active forces of up to 4 N at L4/L5. When standing followed, the L3/L4 IDP calculations (0.31 MPa) correlated very well with the IDP (0.27 MPa) measured *in vivo* by Schultz et al. (1982) for a healthy patient with a slightly lower body weight (63 kg). The prediction also laid in the range of measurements of Andersson et al. (1974) between 0.26 and 0.42 MPa for healthy subjects with body weight ranging between 53 and 77 kg. Indeed, when lying position was simulated before standing, IDP increased substantially and pressure results better reproduced previous *in vivo* studies, given the ability of the disk model to capture the osmotically induced disk turgor. After 8 h of simulated rest, the IDP increased at all different levels by about 0.14 MPa, matching *in vivo* measurements achieved after 7 h of overnight rest (Wilke et al., 1999) for a L4/L5 healthy disk of a subject with similar anthropometric profile. During standing after previous rest, our L4/L5 IDP predictions formally laid within the range of values reported by Sato et al. (1999) but seemed to be slightly underestimated statistically. Interestingly, Rohlmann et al. (2009) intended to simulate indirectly the muscle effects on IVD loads with an osteoligamentous model geometry similar to ours, while they achieved good approximation of the L4/L5 IDP, they systematically understimated the L3/L4 pressures. Hence, effect of model geometry remains to be investigated.

Previous experimental and numerical studies regarding the functional relations that may exist between IVDs and muscles in the lumbar spine have reported controversial results. Wilke et al. (1996) performed *in vitro* tests on cadaveric spines with externally fixed cables that simulated the basic back muscles. They measured the IDP through a pressure transducer with a needle inserted in the center of the NP, and results showed that, in neutral position, muscle forces always increased the IDP by more than 200%. In contrast, Goel et al. (1993) combined FE modeling and optimization approaches, and calculated that muscle presence decreased the IDP in monosegmental osteoligamentous models under flexion. Our model predicted that muscle representation decreased the IDP by up to 9% in standing position without previous night rest, which was due to the posterior mechanical support provided by the fascicles under the action of the anterior body weight. Further calculations showed that previous swelling slightly increased this muscle effect at the uppermost levels (results not shown), suggesting that prestrained muscles became more efficient to restrict the forward rotation of the most cranial vertebra. Such a hypothesis, though, would need to be confirmed with an extended model able to capture larger kinematical changes.

When standing was simulated alone, active forces counteracted the anterior body weight effect and pulled back the spine segment, resulting in fascicle compression. When previous rest was considered, active force predictions revealed that global muscle activity was reduced by up to 68%, while local muscle activity was increased by up to 85%, increasing the effective pull back forces. In particular, the model predicted that overnight swelling led to heterogeneous fascicle activation through the different lumbar levels; during night rest, up to 73, 48, and 24% higher activation was calculated at L3/L4 than at L5/S1 for MF, PS, and lumbar LT, respectively. Increased activation at L3/L4 resulted from the cumulative effect of disks swelling from L5/S1 to L3/L4. According to this higher pre-activation at L3/L5, once standing was simulated, the total loads transferred to the L3/L4 level decreased by more than 60% compared to standing without previous swelling, and global muscle forces decreased causally. Remarkably, eventual fascicle strain was positive understanding with previous swelling, but with lower absolute stretch values compared to the standing case alone. These outcomes suggest that previous swelling might limit muscle strain understanding, while improving the capacity of the fascicles to mechanically stabilize the spine.

Regarding the simulation of body weight, previous studies reported the application of a single vertical load at a point placed anteriorly that represented the center of gravity at L1/L2 (Gardner-Morse et al., 1995; Rohlmann et al., 2006) or at each lumbar level (Zander et al., 2001). In our model, a gravity load was distributed over the model and the resultant of 276 N obtained at L5/S1 stood for about 40% of the total BM for a 70.8 kg subject. In terms of magnitude, this estimation was close to the 260 N reported by Rohlmann et al. (2006) for a 56 kg subject, standing for about 46% of the total BM. The latter authors also reported an effective distance *R*_i_ of 30 mm for the application of a single vertical load at T12/L1 that simulated the body weight, which qualitatively correlated well with our effective *R*_i_ of 41.4 mm at L3/L4. Good agreement was found also with both the 245 N gravity force estimate by Shirazi-Adl et al. (2002) and the distribution of this force per level: 205.6 N (applied between L1/L2–L3/L4), 19.3 N (L4/L5), and 20.1 N (L5/S1 level).

While the outcomes of this study contribute to the first educated exploration of a possible interaction between disk swelling and muscle function, our model has some limitations. Our first intention was to have a 3D representation of the muscle geometry based on MRI data. However, this task was extremely challenging because of the difficulty in distinguishing the different lumbar fascicles. Thus, we have simplified our model and adopted a common use of discrete models to represent the lumbar muscles (Arjmand and Shirazi-Adl, 2006; Gagnon et al., 2011). Nonetheless, in absence of previous swelling, analysis of the PS force components revealed that the volumetric passive stress of the matrix overcame the active stress of the fibers, under simulated standing. Hence, full modeling of the fascicle volume may reveal significant interactions between adjacent fascicles through Poisson’s effects. A common dorsal site at the estimated location of the third rib was chosen to simulate the rostral insertion of the thoracic elements (LTpTh) given the lack of a thoracic cage geometrical model. This simplification affected the lengths of the L4 and L5 fascicles compared to the musculotendon lengths reported by Christophy et al. (2012). Yet, these fascicles remained mechanically inactive in our reduced L3–S1 model, due to both the loads simulated and the zero displacements imposed at the lower bony endplate of the L5–S1 IVD. When simulating standing, we assumed that the axial alignment of L3 and the third rib should be preserved, though this approximation would be more correct if L1 is taken as a reference instead of L3. Therefore, we might expect some overestimation of LTpTh forces in the present L3–S1 model understanding. Nevertheless, given the predominant role of the local muscles and PS fascicles, this limitation should not affect our current model interpretations. As for ILpTh, their contribution in standing was estimated to be about half of that of LTpTh (Arjmand and Shirazi-Adl, 2006). As such, ILpTh omission seemed acceptable for our specific calculations.

For the muscle parameter values definition, our active parameter values were based on the assumption that normalized length ratios are preserved from the sarcomere level to the fiber level (Christophy et al., 2012). A total of eight active muscle parameter values (*C*_CE1_, *C*_CE2_ at Table 1) was used for the entire L3–S1 model as reported elsewhere (Toumanidou et al., 2013). For the passive parameters, a common matching set of four values for all muscle groups was defined giving a total number of 12 muscle values for the entire model. This reduced number of parameters simplifies the choice of values. Martins et al. (1998) used prescribed contractile strains to control the active muscle contraction and they assessed the proposed model through direct definition of the ζ^CE^ value based on previous RB dynamics/optimization analyses. Instead, Ho Ba Tho et al. (2014) used MRI-based strain measurements to simulate different expressions of facial muscles and reported realistic displacement predictions. Indeed, the consideration of a decoupled expression for the muscle contractile strain through the parameter *C*_CE_ (Eq. 9 and 11) is advantageous for calibrations through MRI data obtained for the different lumbar spine muscles. Such incorporation of the CE in the model through strain thresholds allowed for a simplified calculation of the velocity of contraction through the history of ε along the simulations. For the calculation of *v*_max_ based on Hill (1938), at least two additional model parameters would be needed, which would complicate the choice of the parameter values, especially because no relevant data have been reported previously for the back muscles.

Yet, further extension of the muscle model is considered important to incorporate time effects and simulate the influence of dynamic motions involved in activities, such as gait (Krebs et al., 1992), in contrast to the effect of static loads. In particular, the passive properties of skeletal muscles are known to be viscoelastic (Best et al., 1994). Our simulations of standing position did not consider any long-lasting loads. Nevertheless, fascicle deformations during simulated night rest occurred at very low-strain rates, including viscoelasticity would have given a passive muscle stiffness lower than the effective stiffness shortly after standing. Therefore, disregarding muscle viscoelasticity is a limitation of the current model. Interestingly, intrinsic viscoelasticity mostly affects the deviatoric response of hyperelastic materials (Holzapfel, 2000), and active forces during overnight swelling were 2–10 times higher than the deviatoric passive force in the local muscles. Such predominant role of the active force suggested that the simulated effect of previous swelling on stretch-induced muscle activation and posterior segmental stabilization might not be significantly affected by the non-inclusion of muscle viscoelasticity into the model.

To conclude, in this study, a new predictive L3–S1 FE musculoskeletal model of the lumbar spine was developed allowing for exploration of coupling between muscle forces and the non-linear and transient mechanical behavior of the surrounding spine tissues. A remarkable effect of overnight IVD swelling was found on the mechanical role of muscles in standing posture. The results suggested a functional relation between sufficient resting, disk multiphysics, and muscle activation toward optimal mechanical stabilization of the trunk. Despite the difficulty in achieving exhaustive model validation, realistic IDP predictions compared to *in vivo* data supported the ability of the model to generate well-informed analyses of the underexplored relationship between muscle activity and disk biomechanics. The limited parametric dependence of the constitutive formulation proposed for the muscle model was considered advantageous for further calibrations/assessments based on patient MRIs. One step forward would be the consideration of the interaction between muscle mechanical response and degenerated disk properties, as the latter modify the multiphysics disk behavior (Malandrino et al., 2015).

## Conflict of Interest Statement

The authors declare that the research was conducted in the absence of any commercial or financial relationships that could be construed as a potential conflict of interest.

## Supplementary Material

The Supplementary Material for this article can be found online at http://journal.frontiersin.org/article/10.3389/fbioe.2015.00111
