# Corrigendum: Musculoskeletal Modeling of the Lumbar Spine to Explore Functional Interactions between Back Muscle Loads and Intervertebral Disk Multiphysics

**DOI:** 10.3389/fbioe.2015.00163

**Published:** 2015-10-14

**Authors:** Themis Toumanidou, Jérôme Noailly

**Affiliations:** ^1^Institute for Bioengineering of Catalonia, Barcelona, Spain; ^2^Department of Information and Communication Technologies, Universitat Pompeu Fabra, Barcelona, Spain

**Keywords:** constitutive muscle model, lumbar spine finite element model, intervertebral disk swelling, intervertebral disk-muscle interaction, standing, night rest

In Toumanidou and Noailly (2015), the general forms of the second Piola-Kirchhoff and Cauchy stress tensors on Eqs (12) and (16) in Materials and Methods were not reported correctly. This should read as follows:

(12) $$\text{S}=2\frac{\partial U}{\partial \text{C}}=\frac{G}{2}\left(2J^{-2∕3}\text{I}-\frac{2}{3}{\bar{\text{I}}}_{1}^{C}{\text{C}}^{-1}\right)+K\ln J{\text{C}}^{-1}+U_{F}^{\prime }\left[J^{-\frac{2}{3}}{\bar{\lambda }}_{f}^{-1}\left(\text{N}⊗\text{N}\right)-\frac{1}{3}{\bar{\lambda }}_{f}{\text{C}}^{-1}\right]$$

The Cauchy stress was related to the second Piola-Kirchhoff stress by:

(16) $$\sigma =\frac{1}{J}\text{FS}{\text{F}}^{-T}=\frac{G}{2J}\left(2\bar{\text{B}}-\frac{2}{3}{\bar{\text{I}}}_{1}^{C}\text{I}\right)+\frac{K\ln J}{J}\text{I}+\frac{1}{J}\left[U_{F}^{\prime }\left({\bar{\lambda }}_{f}\left(\text{n}⊗\text{n}\right)-\frac{1}{3}{\bar{\lambda }}_{f}\text{I}\right)\right]$$

where ***n*** is the direction of the muscle fibers in the deformed fascicle, $\bar{\text{B}}$ the deviatoric part of the left Cauchy-Green tensor **B**, and **I** the second-order unit tensor.

## Conflict of Interest Statement

The authors declare that the research was conducted in the absence of any commercial or financial relationships that could be construed as a potential conflict of interest.

