# Supplementary material for: Musculoskeletal Modeling of the Lumbar Spine to Explore Functional Interactions between Back Muscle Loads and Intervertebral Disk Multiphysics
Source: Front Bioeng Biotechnol. 2015 Aug 5;3:111. doi: 10.3389/fbioe.2015.00111 (PMC4525063; doi:10.3389/fbioe.2015.00111)
Supplement: Supplementary file 1 [file Data_Sheet_1.DOCX]

***Supplementary Material***

Musculoskeletal modelling of the lumbar spine in order to capture possible interactions between back muscle loads and intervertebral disc multiphysics

**Themis Toumanidou^1,2^, Jérôme Noailly^1,2*^**

^1^Institute for Bioengineering of Catalonia (IBEC), C/ Baldiri Reixac, 10-12, 08028 Barcelona, SPAIN

^2^Department of Information and Communication Technologies (DTIC), Universitat Pompeu Fabra (UPF), C/ Roc Boronat, 138, 08018 Barcelona, SPAIN

*** Correspondence:** Dr. Jérôme Noailly, Department of Information and Communication Technologies (DTIC), Universitat Pompeu Fabra (UPF), Barcelona, Spain, tel: +34 93 542 15 79

[jerome.noailly@upf.edu](mailto:jerome.noailly@upf.edu)

1. **Supplementary Data**

*A. Geometrical details for the muscle fascicle definition*

The muscle architecture of 23 sagittally symmetric fascicle pairs featured three main muscle groups of the lower back: the multifidus (MF), the erector spinae (ES) and the psoas major (PM). For each fascicle, m, of the Erector Spinae, the axial (*X_v_*) and posterior (*X_p_*) lines of actions (Fig. A.1) were resolved in the local coordinate system of the vertebra according to Bogduk et al. (1992a):

$X_{v_{m}}=X_{sag}\cdot\cos\left( \lambda-\sum_{k=1}^{m} \beta_{k} \right)$ **(A.1)**

$X_{p_{m}}=X_{sag}\cdot\sin\left( \lambda-\sum_{k=1}^{m} \beta_{k} \right)$ **(A.2)**

where *X_sag_* the sagittal fascicle projection, $\lambda$ the angle between the fascicle and the long axis of the vertebra, $\beta$ is the intersegmental lordotic angle of the upper segment.

While *X_sag_* and $\lambda$ were directly taken from the literature (Bogduk et al. 1992a), $\beta$ angles were calculated using the L3-S1 FE model to which the muscle geometrical model was coupled. The points of origin of the fascicles were also determined in function of the specific anatomy of the bi-segment lumbar spine model.

*B. Calculation details for the intradiscal pressure (IDP)*

The calculation of the IDP was based on Wilson et al. (2005) and the biphasic theory where, assuming that the tissue consists of an incompressible solid hydrated with an incompressible fluid, the total axial stress component $\sigma_{11}$ (hereafter called intradiscal pressure) in the center of the disc is given by:

$\sigma_{11}=-\mu^{f}I_{11}+\sigma_{S, 11}$ **(B.1)**

where $\mu^{f}$ is the water chemical potential and $\sigma_{S, 11}$ the total solid stress component in the fibre direction without the swelling pressure (0.15 MPa for healthy discs used in this study).

As such, the IDP was calculated respectively in the center of NP at all different IVDs at the end of each simulated activity as given in Fig. 7 and Table 4.

1. **Supplementary Figures and Tables**

## Supplementary Figures


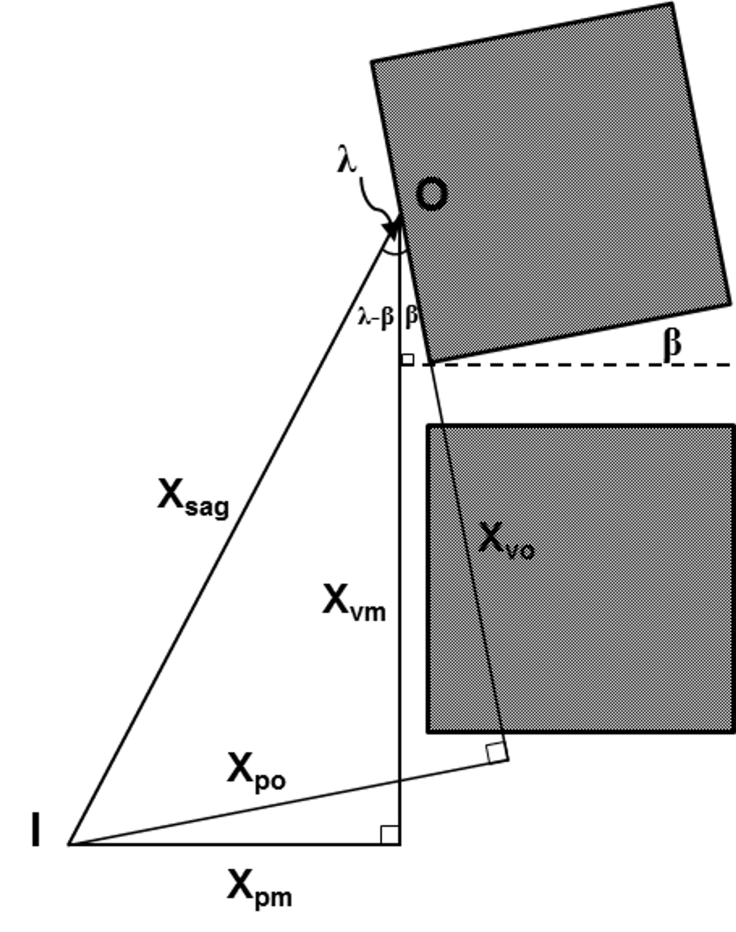


Supplementary Figure 1. A diagram of a motion segment showing the relationship between the axial and posterior (shear) vectors of the fascicle OI on its vertebra of origin and on vertebrae interposed between its attachments sites (O: origin, I: insertion).

References

Wilson W, van Donkelaar C.C. and Huyghe, J.M. (2005) A comparison between mechano-electrochemical biphasic swelling theories for soft hydrated tissues. *J. Biomech. Eng. 127 (1),* 158-165. doi:10.1115/1.1835361
